# On the Treatment of Insanity in Julius Hospital, Würzburg

**Published:** 1858-01-01

**Authors:** Edward Mottley


### ART. VII.—ON THE TREATMENT OF INSANITY IN JULIUS HOSPITAL, WÜRZBURG,

*Under the Management of*

DER HERR HOFRATH DR. VON MARCUS, Senior Physician of Julius Hospital, Ritter des verdienst Orders der Bayrischen Krone, &c. &c.

(Communicated by EDWARD MOTTLEY, ESQ.)

THE following subjects will be treated *seriatim* :—

1st.—*Six Years' Statistics of the Lunatic Wards in Julius Hospital.* By Dr. ERNST SCHMIDT, late Assistant Physician.*

2nd.—*Clinical Lectures of* Dr. VON MARCUS, *on Insanity, during the Session* 1857-8.

*Introduction by the Translator.*—Julius Hospital, in Würzburg, is the magnificent foundation of Julius Echter, Prince-Bishop, and sixty-sixth Bishop of Würzburg, and Duke of Franconia. This eminent prince and prelate, who must be classed with the foremost benefactors of his fatherland, who founded an hospital and a university in Würzburg, and rebuilt or restored twenty hospitals and more than one hundred and twenty churches in various parts of his Principality, was born on the 18th of March, 1545, at Mespelbrun, in the Spessart, about twenty-two miles from Frankfort-on-the-Maine, and died at Würzburg on the 13th of September, 1617, one year before the breaking out of the devastating thirty years' war. He was the last descendant of the time-honoured and illustrious family of Echter von Mespelbrun; and after studying in Belgium and France, finished his education in Rome, where he obtained the degree of Doctor of Civil Law. On the 17th of August, 1570, he was chosen Dean of Würzburg, chiefly through the intercession of Fredrich von Würzburg, the reigning Prince-Bishop, who appears to have foreseen his future greatness; and on the demise of that prelate was elected to the vacant throne on the 1st of December, 1573, being then under thirty years of age. His election was confirmed by the Emperor Maximilian the Second and the Pope; but the ceremony of his installation was deferred until Whitsuntide, 1575, when he was at the same time consecrated priest.

His reign extended over the lengthened period of forty-four years (the average possession of the see by the eighty-three bishops—ending with Carl von Fechtenback, 1808—being about twelve years and four months), "of which," to employ the language of one of his encomiasts, "nearly every day was distin-

* "Sechsjährige Statistic der Ivrenabtheilung des Julius Hospitas," from the Zum Schutze der Irren: by Dr. Ernst Schmidt, a physician of rising reputation, now in America (Chicago).

guished by some important act. His government was characterized by a profuse but judicious liberality in public works. He found his diocese greatly reduced by debt, by the peasant war, and by the defection of great numbers of his subjects, who had embraced the Protestant faith;—churches and hospitals were in ruins, cloisters and monasteries abandoned, and their possessions wasted and destroyed. A mind less vigorous, and a less skilful administrator, might have viewed its condition with despair, and turned from the task of restoring its shattered finances. The first act of his reign was to limit and control the disorder and extravagance of his court, and he lived after his election in such retirement and seclusion, that serious doubts were entertained of his capacity for the good government of his diocese; but these fears were soon dissipated, when one great work after another, matured in this retirement, was carried into execution with extraordinary perseverance and success. Resolved to dedicate his energy and ability to the see of Würzburg, he refused the Palatinate and Archbishopric of Mayence, to which he had been elected by the Chapter in 1582. The two great works of Julius, and by which his name has become historical, are the University and Hospital of Würzburg. Struck by the almost total want of institutions for the benefit of the poor, while the nobility and clergy were amply provided for, he "resolved," as stated in the charter deed, "to endow a Spittal for all sorts of poor, sick, and impotent persons requiring medical, surgical, and other necessary assistance; for abandoned orphans, pilgrims, travelling labourers, and other necessitous persons." The foundation-stone of the original building was laid by Julius on the 12th of March, 1576, and was opened on the 10th of June, 1580. The extensive circuit of the building contained a mill, a slaughter-house, a large garden, baths, and an "inn" for the reception of poor wayfarers, who were there entertained for a day and a night; and everything necessary and convenient for the reception of the poor and infirm belonging to the Principality (for our poor brothers and sisters, as stated in the charter), for sick persons—with the exception of patients with disgusting and contagious diseases—orphans, pilgrims, and labourers, when sick; such persons to receive and be supplied with such clothing, meat, and drink, and necessaries, as the income of the foundation would allow. The last clause of this document threatens "that whoever deprives the hospital of anything—that is to say, the poor and abandoned orphans, and the sick—shall be accused as perverters of the charity, and dishonourers of God's glory, before His judgment-seat on the last day." In one remarkable instance, this threat operated very successfully. When Gustavus Adolphus, after plundering the town and university of Würzburg, visited the hospital with the intention of emptying its then well-filled coffers, the reverend principal of the hospital read the concluding paragraph to the Swedish King, who, being much struck with its import, drew back, saying, "I will have nothing to do with this priest in the other world," and left the hospital with its treasury untouched. At the time of its foundation, the property of the hospital was estimated at 500,000 florins (42,000*l.* nearly); but the accumulation of nearly 300 years of good management has increased it to 6,000,000 florins (500,000*l.*), consisting chiefly of estates in Bavaria and Baden. The duties and benefits have increased in an equal degree, and the single physician and chaplain, and the 120 inmates of the founder's time, have augmented to three senior physicians* and five assistants, an apothecary with two assistants, a house-surgeon, a chaplain and two vicars, with about 900 inmates of all classes, consisting of pensioners (pfründners), sick persons without distinction of country or religion, and an asylum for the reception of curable insane patients. As a medical school, it possesses a European reputation.

The original building has yielded to time, and to a destructive fire which occurred at the close of the eighteenth century; the present noble structure bears the date 1791. Such is the flourishing condition of an institution whose founder was, by his contemporaries, deservedly called great. If, in his zeal for his religion, he was at times severe, his faults were those of the times in which he lived; his virtues were his own. The general condition of the hospital not being the object of this article, we will limit our observations to that division dedicated to the relief of insane patients, under the benevolent superintendence of Dr. von Marcus, and take for our guide Dr. Ernst Schmidt's "Six Years' Statistics of the Insane Wards of Julius Hospital," who says:—

"It is evident that for more than 200 years the insane in Julius Hospital were treated merely as pensioners, and the first weak attempt to introduce a rational system of psychology (psychiatrik) took place about the year 1780; the locality for the then patients being the same as that now used for the incurable—viz., a large secure vaulted hall, on the ground floor of one of the wings connecting the principal buildings. It may be truly considered as one of the first institutions of its kind in Germany; and it was, indeed, somewhat more friendly and humane than many others—at least, we are so led to conclude, from the examples of similar buildings that are existing at the present day—for example, the famous Narrenthurm, at Vienna. At the commencement of the

* The appointment of the senior physician is honorary; each receives a nominal fee of 300f. (25*l.*) yearly, the salary of Upsilius, the first physician.

present century, the number of patients was somewhat above 30, which soon augmented to 40, which was at that time considered as the normal number, and only exceeded in cases of pressing necessity. The proper separation of the insane into curable and incurable patients appears to have taken place between the years 1785-90—at least, such is the information derived from the account of Dr. Müller, the senior physician, whose book on the treatment of the insane was published in the year 1824. This account extends from 1796 to 1823, and gives 528 patients as being received—viz., 258 men, and 270 women. During this time, only 21 patients, on the average, were annually received; but the number was constantly increasing, 15 being received during his first year of office, and 25 the last. What he relates of the proportion of his successful cases must be received with some reserve, as it is contradicted by the more sober computations of the present day. Dr. Müller gives 292 patients, or 56 per cent., as perfectly restored; and 62, or 11 per cent., as improved; while at the present day, 40 per cent. perfectly restored must be taken as a very favourable result. Under the next physician, Dr. Schönlein (the present chief physician to the King of Prussia), about 400 curable patients were received between the years 1823 and 1833. Materials to show the result of his practice are not to be found. Towards the end of the year 1833, Dr. von Marcus was appointed senior physician of Julius Hospital, professor of clinical medicine, and director of the internal management of Julius Hospital, as regards the insane and the pensioners. What he accomplished as professor of clinical medicine may be given in another place; what he effected as senior physician—his earnest zeal for the adoption of all contemporary improvements, and his disinterested exertions for the welfare of the noble institution to which he was appointed principal—will give his name a lasting endurance in its annals.

"Above all, we have to thank him for the arrangement and improvement of the lunatic wards as they now exist, and which need not shun a comparison with any institution especially dedicated to the reception of insane patients—notwithstanding many deficiencies which, in old buildings first appropriated to this purpose, were with us not to be obviated, and which are demanded by the multitudinous requirements of the present day, and not unfrequently carried to excess by the asylum physician.

"During the first nine years of the direction of Dr. von Marcus, the number of applicants so increased, that frequently, by improper crowding, 60 patients were received in one year—until the year 1841, when he introduced those extensive alterations and improvements which will allow the reception of from 90 to 100 patients. Two extensive wings, perfectly adapted for the purpose, are appropriated for curable patients." [For cleanliness, neatness, and cheerful aspect, these apartments are everything that can be desired. Every patient has a separate bed, furnished with curtains—and the whole range of apartments have the appearance of a well-ordered English academy.—E. M.]

"All the cells for dangerous patients are floored and wainscoted with wood, and the approaches to the stoves and windows painted of a light-green colour, so that these cells make no unfavourable impression. Some handsome isolated apartments, for private patients, are also provided. For extremely noisy and raving patients, fitting cells, capable of being easily warmed (heitzvare), are erected in a court where the occasional noise cannot be heard by the convalescent patient (a precaution not always sufficiently attended to in asylums). A retired part of the garden is appropriated to gymnastic exercises, skittles, &c., and which, with its summer-house, affords on fine evenings an agreeable spot for recreation and recovery. Opportunity for manual labour, so far as it is desirable as an auxiliary for recovery, is provided, under the superintendence of an attendant, in the wood-house. The numerous and well-chosen attendants render the use of coercion chairs and strait-jackets only necessary in extreme cases. The new steam douche and shower-baths (the latter often of the greatest service to insane patients) leave little to be wished; indeed such satisfactory arrangements are hardly to be met with in many larger establishments.

"Great importance being attached to the quantity and quality of the food for patients in asylums, we will give the Diet for the Sick, or No. 2 Diet Table:—

"No. 5.—Dinner—$\frac{4}{4}$, or full portion—meat soup, with white bread; $\frac{1}{2}$lb. beef, with vegetables.

"Supper—Soup, with $\frac{1}{2}$lb. veal or calf in meat.

"Breakfast—A portion of soup, with white bread.

"Bread—For the whole day, 14ozs. of rye or 11ozs. of wheat bread.

"Wine—Each patient receives as much wine of the second-class as the senior physician thinks necessary.

"Extra food and wine can be given by the permission of the three senior physicians; and wine of the first-class."

"The above Table is reduced $\frac{3}{4}$, $\frac{2}{4}$, $\frac{1}{4}$, at the discretion of the medical attendant; fruit, coffee, poultry, are also frequently given.

"Immediately after the completion of the improvements, the number of applicants increased, and the present more exact statistics show the capabilities of receiving 100 patients annually.

"In a financial point of view, the admission of curable patients to the hospital is extremely favourable. All persons admissible as pensioners are received as patients—that is to say, all the poor of the former Principality of Würzburg, and now of the whole of the circle of Lower Franconia, are received free of charge, with the exception of the necessary clothing, which must be provided by the parish (tleimath gemeinde) to which the patient belongs. But even this regulation is not insisted upon when the parish is notoriously poor.

"Patients with independent property are admitted upon the payment of 30ks. (10*d.*) daily; and when they require a separate apartment and special attendant, 1 florin (1*s.* 8*d.*)

"When we state that, between 1798 and 1854, more than 2500 curable patients have been received—nearly 2000 gratis—and if wetake into account 250 incurable patients received as pensioners for the whole term of their life, it does not require another word to prove that the division of Julius Hospital appropriated to the reception of insane patients has acquired an historical right to the gratitude of the circle of Lower Franconia.

"Consulting the more exact statistics of our own time, from 1848 to 1854, we find that the reception of curable patients during that time (including a surplus from 1847-1848) amounted to 512—viz., 287 men, 225 women; the re-admissions during that period being—

| | | | | | | |
|---|---|---|---|---|---|---|
| Men . | 1 - | 6 times | | Women . | 16 - | twice |
| " | 2 - | 4 " | | | | |
| " | 3 - | 3 " | | | | |
| " | 25 - | twice | | | | |

"The number of individuals is therefore reduced to 454—or 245 men, and 209 women (54 and 46 per cent.) Singly considered, in four years the men, and in two years the women predominated. In order to form a judgment at what season of the year the greatest number of cases occur, requires a greater exactness of observation of the previous and earlier symptoms (anamnese) than unfortunately is generally the case; as we know that not only the laity, but unfortunately the faculty, do not sufficiently distinguish between cause and effect—essentials and non-essentials—putting aside the intentional deception that is too frequently practised by the patient's friends, and, alas! often by the medical attendant, with regard to the time the patient has been affected previous to being brought to the asylum. We will, however, take 132 cases, on which no doubt rests as to the date of the attack:—

| | | | |
|---|---|---|---|
| January - 14. | April - 12. | July - 9. | October - 8. |
| February 11. | May - 11. | August 10. | November 7. |
| March - - 13. | June - 13. | Sept. - 14. | December 10. |

"This Table contradicts the assumption that has prevailed nearly undisputed since the statement of Esquirol—viz., that the greatest number of cases occur in the hot months—as it must be admitted that a comparison of many thousand examples show a marked increase of attacks of insanity from January to July, and a corresponding decrease to the following January—the latter month giving 6·5 per cent. to the 10·5 per cent. of July. These proportions appear pretty correct for the south of France, Italy, and similar climates, whose winters resemble those of our latter autumn. So with us, to the more wealthy, who cannot be so perfectly and continuously protected from the heat of summer as from the cold of winter, the summer months may be equally dangerous; but for the greater part of our country population, and the poorer classes, the injurious effects of winter are greater than the highest summer temperature, which can be readily understood when we consider the more than tropical heat our peasants and labouring classes maintain in their apartments, which serve at once for living and sleeping rooms—where they cook for their families, and prepare food for cattle; this extreme heat, suddenly alternated with the severe cold of the Bavarian winter, too often accompanied with want of sufficient clothing, protection for the feet, and insufficient nourishment—conditions upon which the results of the accompanying Table may be grounded.

"The period of life which most predisposes to mental alienation has always been one that has occupied the attention of the statistical writer. Esquirol maintained that, with men, from thirty to forty, and with women from fifty to sixty years, were the most dangerous. I must acknowledge that to me this statement has been one that, with reference to our social and natural relations, appears to be little founded upon reason. In fact, we have quite a different result from more than five hundred cases. [The re-admissions must be included in the number, because predisposition at a peculiar age will also render the re-admissions more numerous.] The following Table gives the ages:—

| | Before 15 | 15 25 | 25 35 | 35 45 | 45 55 | 55 65 | 65 75 | After 75 |
|---|---|---|---|---|---|---|---|---|
| OF MEN. | | | | | | | | |
| | 1 | 53 | 77 | 68 | 58 | 22 | 6 | 2 |
| Per cent. | $\frac{1}{3}$ | $18\frac{1}{2}$ | 27 | $23\frac{1}{2}$ | 20 | 8 | 2 | $\frac{2}{3}$ |
| OF WOMEN. | | | | | | | | |
| | 2 | 41 | 90 | 58 | 35 | 17 | 2 | 0 |
| Per cent. | $\frac{4}{5}$ | $16\frac{3}{4}$ | $36\frac{3}{4}$ | $23\frac{2}{5}$ | $14\frac{1}{2}$ | 7 | $\frac{4}{5}$ | 0 |

Consequently, with us the greatest number of cases occur, with both sexes, between the ages of twenty-five and thirty-five, at which period of life the human being attains the zenith of his or her mental and physical powers; after which time, except in rare instances and extraordinarily gifted individuals, those powers become more languid, and a greater passivity predominates over all the functions. This is also the period when, in most cases, the individual commences an independent existence, and loses the support of his family. Now begins, with most persons, the greatest exertions; the time when the passions and affections expand, under the influence of the meridian hour; the time when the mental powers are put to the severest trials, and become steeled and invigorated, or, alas! by disappointed hopes, unfortunate speculations, or by powerful interruptions of the full tide of human passions, too often shattered and destroyed for ever! After this period, the per centage of the cases suffer a marked decline—slower with the men than with the women, whose decline, corresponding with their earlier development and maturity, is equally premature. The few cases of insanity which occur after sixty years of age, with perfectly healthy individuals, mostly belong to dementia senelis and apoplectica, arising in most instances from pathological change. As regards sex, with us, as in most instances, the male patients predominate; yet we can affirm with the greatest truth, that an authentic statement of the difference of numbers between the two sexes requires a much more extended computation over different countries than we have at present. Of the men, 103 were unmarried, and 142 married; of the women, 161 unmarried, and 48 married. Of the men, the greater number, nearly three-fifths, were married; whilst with the women, more than three-fourths were, at least in the world's eye, "withering on the virgin thorn." With the men, masturbation was unhappily proved with more than one-third of the patients; with the women, the proofs are more difficult. In no one instance (at least, with the men) was want of sexual chastity (Seschlects befriedigung) presumed to be the cause of mental alienation."

On the all-important subject of hereditary predisposition, or inheritance (Erblichkeit), Dr Ernst Schmidt says:—

"Hereditary predisposition is proved in more than three-fifths of all cases, to which more than two-fifths to the so-called direct, and one-fifth to indirect, must be attributed. Fortunately, however, the entire doctrine of inheritance begins to be subjected to a more searching criticism, and we must remember in how many cases very different conditions than those of the so-named inheritance are the cause of disease in members of the same family. This much is certain, that in cases of well-proved direct inheritance, but very few patients have ever perfectly recovered."

Dr. Ernst Schmidt now gives the annexed tabular view of the 512 cases treated in Julius Hospital between the years 1848 and 1854:—

| Form of disease. | No. | Cured. | | Improved. | | Uncured. | | Dead. | | Remaining. | Average Age. |
|---|---|---|---|---|---|---|---|---|---|---|---|
| | | | Time in Asylum. | | | | | | | | |
| Pure Hallucination. | | | | | | | | | | | |
| Men | 1 | 1 | 6 wks. | ... | ... | ... | ... | ... | ... | ... | 52 |
| Hypochondria. | | | | | | | | | | | |
| Men | 8 | 2 | 2 mon. | 2 | ... | ... | ... | 3 | 3 mon. | 1 | 39 |
| Women | 2 | 2 | ... | ... | ... | ... | ... | ... | ... | ... | 32 |
| Hysterico. | | | | | | | | | | | |
| Women | 2 | ... | ... | ... | ... | 1 | 22 m. | ... | ... | 1 | |
| Melancholia. | | | | | | | | | | | |
| Men | 68 | 30 | 3 mon. | 19 | 4 mon. | 9 | 8 mon. | 4 | 3½ m. | | 34 |
| Women | 91 | 48 | 4½ m. | 20 | 6 mon. | 11 | 11 m. | 8 | 4 mon. | 4 | 33 |
| Mania and Delirium. | | | | | | | | | | | |
| Men | 87 | 52 | 3½ m. | 14 | 6 mon. | 6 | 8 mon. | 11 | 6 wks. | 4 | 35 |
| Women | 53 | 35 | 4½ m. | 7 | 9 mon. | 6 | 18 m. | 3 | 1 mon. | 2 | 31 |
| Eroto & Nymphomania | | | | | | | | | | | |
| Men | 3 | 1 | 3 mon. | 2 | 1 mon. | ... | ... | ... | ... | ... | 29 |
| Women | 17 | 3 | 3 mon. | 3 | 8 mon. | 10 | 15 m. | ... | ... | 1 | 32 |
| Dementia. | | | | | | | | | | | |
| Men | 82 | 9 | 3 mon. | 22 | 7 mon. | 35 | 8 mon. | 8 | 2 mon. | 8 | |
| Women | 52 | 4 | 6 mon. | 10 | 10 m. | 29 | 12 m. | 4 | 8 mon. | 5 | |
| Stupiditas. | | | | | | | | | | | |
| Men | 17 | ... | ... | 2 | 6 mon. | 14 | 6 mon. | 1 | ... | 30 | 30 |
| Women | 6 | ... | ... | ... | ... | 6 | 7 mon. | ... | ... | ... | 26 |
| Delirium Tremens. | | | | | | | | | | | |
| Men | 15 | 12 | 1 mon. | ... | ... | 1 | 2 mon. | 2 | 1 day. | ... | 38 |
| Women | 1 | 1 | 1 mon. | ... | ... | ... | ... | ... | ... | ... | 43 |
| Epilepsy. | | | | | | | | | | | |
| Men | 5 | 1 | 2 mon. | 2 | 2 mon. | 2 | 5 mon. | ... | ... | ... | 35 |
| Women | 1 | ... | ... | ... | ... | 1 | 5 mon. | ... | ... | ... | 10 |
| Chorea with Delirium. | | | | | | | | | | | |
| Men | 1 | 1 | 6 wks. | ... | ... | ... | ... | ... | ... | ... | 10 |
| Men | 287 | 109 | ... | 63 | ... | 67 | ... | 29 | ... | 19 | ... |
| Women | 225 | 93 | ... | 40 | ... | 64 | ... | 15 | ... | 13 | ... |
| | 512 | 202 | ... | 103 | ... | 131 | ... | 44 | ... | 32 | ... |

In the remarks appended by Dr. E. Schmidt to the Table, he says:—

"Our most numerous form of disease was melancholia—viz., 40 per cent. of all the women, 23 per cent. of the men. Mania and delirium (tobsucht) is represented by 30 per cent. men, and 23 per cent. women. With dementia, including dementia senelis, 28 per cent. men, and 23 per cent. women. The most favourable result as regards cure, considering the number of cases of each peculiar form of disease, is delirium tremens—80 per cent.; then maniacal female patients, of whom 66 per cent. left the asylum cured; maniacal male patients, 59 per cent. Melancholia gave a more favourable result for women than men; of the first, 52, and of the latter, 44 per cent. cured. The proportion of fatal cases was equally unfavourable for the men, of whom 10 per cent. died of the whole number received, and of the women nearly 7 per cent. The form of disease most fatal was hypochondria, which exhibits with the men, 37 per cent.; with the women, melancholia, nearly 9 per cent. With male maniacs, above 12 per cent.; but with women, hardly 6 per cent. died. On the average of all the cases, the deceased were the shortest time in the asylum; and the patients the longest under treatment were the maniacal women, who were dismissed uncured after eighteen months' treatment; the nymphomaniste, 15 months; one hysterische, 22, &c. The most speedy recovery was with delirium tremens—4 weeks, average; the latest, with melancholische women—4½ months. However we may be satisfied with the result of the treatment at Julius Hospital, we cannot conclude this statistical voucher without forbearing to mention the great evil in treatment of lunatics, and whose sin it is that we cannot show a result still more favourable—we mean, the neglect in not bringing the patient to the asylum at an earlier period. We will employ 220 cases, where the correctness of the time of the duration of the disease previous to the reception in the asylum admits of no doubt:—

| Duration of disease previous to reception in the asylum. | No. of cases. | Of which were healed. |
|---|---|---|
| 1 to 3 months. | 39 | 25 = 64 per cent. |
| 3 to 6 ,, | 46 | 20 = 43 ,, |
| 6 to 12 ,, | 48 | 16 = 33 ,, |
| 1 to 3 years. | 87 | 15 = 17 ,, |
| | 220 | 76 = 34 per cent. |

"These numbers require no commentary, and show at the first look where the foulest spot of our present management lies (irrenuesens). Its removal would diminish the inmates of our asylums one-third. . . . How and in what manner Hofrath Dr. von Marcus rendered our institution available for the purpose of clinical instruction, is too well known and acknowledged to require more than a passing notice here. Since the year 1841, Dr. von Marcus rendered access to the patients practicable, in the form of clinical visitations. A practical course of clinical psychiatria commenced (eine werkliche psychiatrische klinic), and that has been rewarded by a numerous and zealous auditory."

Dr. Ernst Schmidt then gives the following cases, as some of the most interesting that occurred during 1848-54. Of chorea St. Viti, accompanied with delirium, we have a case of great interest:—

"1. A boy, ten years of age, healthy, and without family taint, one day walking with his mother, stooped to pick up what he thought a handsome stick lying in the road. The adder (for such it was), when seized, wound round his arm without hurting him, and escaped. The boy cried aloud, trembled, and in a few days presented a perfect type of chorea St. Viti. The delirium that supervened wore a religious character. Treated with nutritious food, cheerful amusements, and the employment of Valerian iron. Perfectly cured in less than two months.

2. "R——, colour manufacturer; healthy, with an inclination to spirits. In domestic and financial relations, not fortunate. In 1849, employed himself zealously with the return of members to Chamber of Deputies. Attempted electioneering himself, and thought seriously of becoming a deputy. His imagination soon passed the limits of reality—he firmly believed that he was chosen deputy, for the purpose of being named President of the Chamber. He came to Würzburg in consequence, to fetch his patent from the Government. Felt confident of becoming Duke of Franconia, in which case he promised us his favour and protection. Cured in three months.

"3. S. B——, twenty-one years old; slight, without hereditary predisposition. Often chlorotic; suffered from menorrhagia. Was one day offended, and her modesty outraged, by a young man suddenly exposing her person during the time of her menstruation. She was suddenly seized with convulsions; a few hours after, with delirium. In a few days, mania supervened. She raved uninterruptedly; was uncleanly and immodest in the highest degree; digestive powers impaired. Cured in eight months.

"4. S. B——, twenty years old, well formed, and powerfully built, without the slightest disposition to hereditary predisposition, had recovered in her eleventh year from an attack of typhus; menstruated at eighteen, irregular, but at the time of her attack regular. In the middle of July, S. B—— fell asleep in a warm bath, and slept in water constantly decreasing in temperature, five hours before she was discovered; in the evening of the same day mania appeared, with ideas of the most exalted character; raves, and becomes dangerous to approach. Four weeks after her attack she was brought to the asylum, and left it in four months perfectly healed.

"5. F——, twenty-two years old, Protestant schoolmaster, healthy, strong constitution; went over to the Catholic Church, and will dedicate himself to the priesthood—felt great anxiety about the validity of the Protestant baptism; doubt its Christianity, and could not be persuaded to the contrary. The day before the one appointed for his full reception into the Catholic Church, he heard a voice which commanded him to make a trial of his being in the grace of God: he obeyed, and sprang out of his window, two stories high, to the court below, and received no injury. He employed himself zealously in prayer, on returning to his room, to be able to resist all temptations of the devil; but the voice continued to follow him, and he obeyed a command to leap from the wall of the fortress into the fosse below, which was fortunately a mere swamp; here he was found torn and bruised, and quite speechless. After four weeks' residence in the asylum, he was so far restored from his demono-melancholia that his friends removed him from the asylum: no relapse.

"6. S. M——, thirty-one years old, servant, single; healthy, without hereditary predisposition; mother of two healthy children—regularly menstruated—suddenly was attacked with religious melancholy on the unexpected death of her father—believed that the wicked would prevail over the good, but she would remain true to her Saviour; and as a propitiatory sacrifice exarticulated the upper joint of her little finger. She was brought to the asylum; suffered an attack of typhus; left in less than three months, perfectly restored, mentally and bodily.

"7. F. B——, forty-five years old, still menstruated regularly; mother of four healthy children; healthy. Insanity was proved in one member of her family—father's brother; was suddenly attacked with religious melancholy, after hearing twenty sermons consecutively from the Jesuit Missionaries; was holy and in a state of grace—and will intercede with God for a fallen world—screamed and raved, and at last bit off an inch and a half of her tongue, which only hung by a few ligaments; the lesion was secured as well as possible, but gangrene supervened; the end of the tongue fell off, and she died in a few days.

"8. J. H——, thirty-nine years old, married, healthy, without direct or indirect predisposition; obtained permission to establish a dyehouse in W——; made the necessary arrangements, carrying on the business with good success, till the authorities forbade the dye refuse to run into the stream that passed by the dyehouse, as the fish suffered in consequence. H—— remonstrated on very reasonable grounds—in that this condition should have been known before he was allowed to erect his premises, and that the revocation of his licence would ruin his business. His remonstrances were without effect; angry words were exchanged; and H—— was imprisoned forty-eight hours for slandering an official person (Amssehren beleidigung). From this time he evinced great irritability, without his friends perceiving any symptom of insanity. When he was informed by a legal officer of a second unfavourable decision against him, he seized an axe, and ran to the office of the magistrate, who was necessitated to escape into an adjoining room. J. H—— attacked the massive door with great violence, and drove the axe with such force into the wood as fortunately rendered him unable to extricate it; he then ran away. Some days after, he allowed himself to be taken willingly to prison by the gensdarmerie, prayed aloud, and did not recognise the magistrate. Two days after, by the advice of the official physician, he was removed to the asylum of Julius Hospital; mania ensued. Cured in six months, and remained perfectly sane for two years. Then H—— began again to demand compensation for the loss of his business, which produced no result, when he again attacked the magistrate in a paroxysm of rage. Captured, he made his escape from his guards, and, after four days' wandering, came himself to the asylum to beg for admission. He was less excited than in his first attack, but suffered from pronounced confirmed delusions—*e.g.*, the Virgin appearing to him, and comforting him by promising to punish his tormentors. Intelligence, thought, and will, quite changed; and his body wasted by sleepless nights, disturbed digestion, continued restlessness. However, after six months' quiet, and restoratives, he became convalescent, and had no relapse."

In our next number, the first of Dr. von Marcus's Lectures will appear.

